# Marked Point Process Secretory Events Statistically Characterize Leptin Pulsatile Dynamics

**DOI:** 10.1210/jendso/bvae149

**Published:** 2024-08-29

**Authors:** Qing Xiang, Revanth Reddy, Rose T Faghih

**Affiliations:** Department of Biomedical Engineering, Tandon School of Engineering, New York University, New York, NY 11201, USA; Department of Biomedical Engineering, Tandon School of Engineering, New York University, New York, NY 11201, USA; Department of Biomedical Engineering, Tandon School of Engineering, New York University, New York, NY 11201, USA

**Keywords:** leptin, bromocriptine, generalized inverse Gaussian, Akaike's information criterion, Kolmogorov-Smirnov plot, quantile-quantile plot, statistical signal processing

## Abstract

Recent studies have highlighted leptin, a key hormone that regulates energy intake and induces satiety, due to the worldwide prevalence of obesity. In this study, we analyzed plasma leptin measurements from 18 women with premenopausal obesity before and after bromocriptine treatment. By using underlying pulses recovered through deconvolution, we modeled the leptin secretory pulses as marked point processes and applied statistical distributions to evaluate the dynamics of leptin, including the interpulse intervals and amplitudes of the secretion. We fit the generalized inverse Gaussian and lognormal distributions to the intervals and the Gaussian, lognormal, and gamma distributions to the amplitudes of pulses. We evaluated the models’ goodness of fit using statistical metrics including Akaike's information criterion, Kolmogorov-Smirnov plots, and quantile-quantile plots. Our evaluation results revealed the effectiveness of these statistical distributions in modeling leptin secretion. Although the lognormal and gamma distributions performed the best based on the metrics, we found all distributions capable of accurately modeling the timing of secretory events, leading us to a better understanding of the physiology of leptin secretion and providing a basis for leptin monitoring. In terms of pulse amplitude, the evaluation metrics indicated the gamma distribution as the most accurate statistical representation. We found no statistically significant effect of bromocriptine intake on the model parameters except for one distribution model.

Obesity, characterized by excessive fat accumulation, has become a global epidemic with profound health implications [1]. In women, obesity is associated with menstrual perturbations and infertility [2]. For example, a study examined different weight groups of women and found that the group with obesity had the least implantation rate (26.4%) and pregnancy rate (37.9%), whereas the lean, normal weight, and overweight groups had average rates of 31.7% and 44.0%, respectively [3]. Genetics, feeding practices, and sleep health all play important roles in potential obesity development [1]. Leptin, a vital hormone regulating body energy and food intake, is also closely linked to obesity. While body weight is also associated with other hormones such as insulin and ghrelin [4], studies have demonstrated a clear association between plasma leptin levels and body fat percentage [5, 6]. Due to its ability to inhibit hunger and induce satiety, leptin has been considered an important factor in treating obesity. However, the excessive amount of leptin in obese individuals suggests potential leptin resistance [5], and the development of such resistance remains unclear [7]. Leptin is primarily secreted from adipose tissue distributed throughout the human body, including subcutaneous and visceral adipose tissues [8]. Studies have indicated that leptin secretion involves both circadian and ultradian rhythms [9, 10, 11]. Additionally, research has suggested sex-based differences in leptin circulation [12, 13, 14, 15]. Therefore, the underlying mechanism of leptin secretion is complex, and its pattern is challenging to decipher. Despite these challenges, recent studies estimated leptin secretory pulses using signal processing techniques [16, 17]. They identified sequences of leptin secretory events through deconvolution of measured plasma leptin levels in women with obesity and compared the reconstructed leptin levels over time with the measured data. The results revealed subject-dependent variations in the number of pulses within 24 hours. This pulsatile pattern of leptin secretory events is also observed in other hormonal secretions, such as cortisol [16].

We aimed to further analyze the underlying pulses using established methods. In a 2020 study of electrodermal activity (EDA), Subramanian et al [18] used point processes to characterize the pulse events in terms of timing. They fit the generalized inverse Gaussian (GIG) and lognormal probability distribution models to the measured EDA data. Building on this approach, our study applied the same models to the extracted leptin secretory pulses, to gain a better understanding of leptin secretion characteristics. Similar to EDA, leptin secretion is pulsatile [16] and can be characterized by the timings and amplitudes of its underlying secretory pulses. As noted, leptin is secreted into the blood from various body parts, primarily from adipose tissue, and its secretory pulse events are relatively sparse [16, 18]. Before secretion, leptin synthesis is affected by various factors including fasting, diets, and other hormones [19]. Despite the complexity of the synthesis process, we applied the models in the EDA study to characterize the interpulse intervals of leptin secretion based on the similarity in the pulsatile nature both of leptin and sweat secretion. We aimed to test the performance of these models in a new and seemingly more difficult case, where the observation time is shorter and there are fewer secretory pulses, as well as possibly revealing new characteristics of leptin secretion. To accompany the modeling of intervals, we also applied Gaussian, lognormal, and gamma models to study the amplitudes of the pulses for further observations. For the evaluation of the estimated models’ fit, we applied Akaike's information criterion (AIC) for both the interval and amplitude models. In addition, we also made Kolmogorov-Smirnov (KS) plots and computed KS distances for the interval models as another way of evaluation. For the amplitude models, we used quantile-quantile plots (Q-Q plots) to compare them.

We also compared differences in the parameters of the distribution models before and after treatment of bromocriptine, a dopamine agonist frequently used to treat disorders of the neuroendocrine system [20]. Through the framework of our marked point process analysis, observing changes in parameters for both interpulse intervals and amplitude modeling could serve as an effective tool for analyzing a change in hormone dynamics due to the effect of a drug or illness.

## Materials and Methods

### Data Set

We used leptin data collected by Kok et al [21, 22, 23] in their previous clinical studies. The participants were 18 healthy premenopausal women with obesity (body mass index 30.1-40.5, aged 22-51 years with mean age = 37.5 *±* 1.7) who were not under the influence of any medication or drugs. Individuals with acute or chronic disease, depression, head trauma, habits of smoking or alcohol consumption, recent transmeridian flights, nightshift work, weight change, blood donation, or participation in another clinical trial were excluded. To minimize potential confounding factors, the data were gathered during the early follicular phase of their menstrual cycles, and all participants maintained a eucaloric diet and adhered to the same sleep schedule, from 11 Pm to 7:30 Am. The study period spanned 24 hours, from 9 Am to 9 Am the next day, following 7 days of placebo treatment. Hormone concentration levels were measured from blood samples using radioimmunoassay, which has a detection limit of 0.5 ng/L, at 10-minute intervals throughout this period [24]. Four weeks later, the same protocol was repeated with the same women. However, this time, each participant received a 2.5-mg dose of bromocriptine twice daily for 7 days to assess the effects of bromocriptine.

In their previous study, Reddy et al [17] deconvolved these plasma leptin measurements to understand the underlying secretion of leptin. Specifically, they extracted the timings and amplitudes of leptin secretory pulses throughout the duration of the recording while considering physiological constraints of leptin secretion such as its pulsatile and sparse nature. There are normally 20 to 50 secretory pulses in 24 hours, and the pulses are discrete [16]. They modeled the leptin secretion process as a state-space model, the details of which are presented in later sections. To perform the deconvolution and recover the leptin pulses, the parameters of the model are solved using the FOCUSS + algorithm and generalized cross-validation. The details of this method are stated in [16, 17, 25]. For the purpose of this study, we applied our statistical models to these extracted pulses for each of the 18 participants.

### Distribution Models

The GIG distribution has been widely used for modeling neural spike trains [26]. Neuronal behaviors are often modeled using random walk models, reflecting their well-known integrate-and-fire mechanisms [27]. In these models, the interspike intervals represent accumulation times, with spikes occurring when a fixed threshold is reached. The density of these interspike intervals in such diffusion processes typically follows the inverse Gaussian distribution [28]. Similarly, EDA, indicative of sweat secretion from sweat glands, exhibits a comparable pulsatile pattern [18]. Therefore, the generalized inverse Gaussian formula, more versatile than the simple inverse Gaussian and including both gamma and exponential models, is used to model EDA's interspike intervals [18]. Additionally, the lognormal model, another prevalent choice for modeling interspike intervals in point processes, is also employed [26].

In our study, we applied the GIG models to leptin pulse events, hypothesizing that the lengths of the interpulse intervals follow a similar distribution to that of the interspike intervals. We also considered the lognormal model as a potential best fit. However, we did not use a one-parameter exponential model due to its demonstrated ineffectiveness in the EDA study [18]. With only one parameter, the exponential model lacks the flexibility required to accurately represent processes like leptin secretion. Intuitively, we did not anticipate the peak of the interpulse intervals distribution to be near zero. Therefore, applying the exponential model to the interpulse intervals distribution would likely result in statistically significant discrepancies near zero.

**Figure 1.**
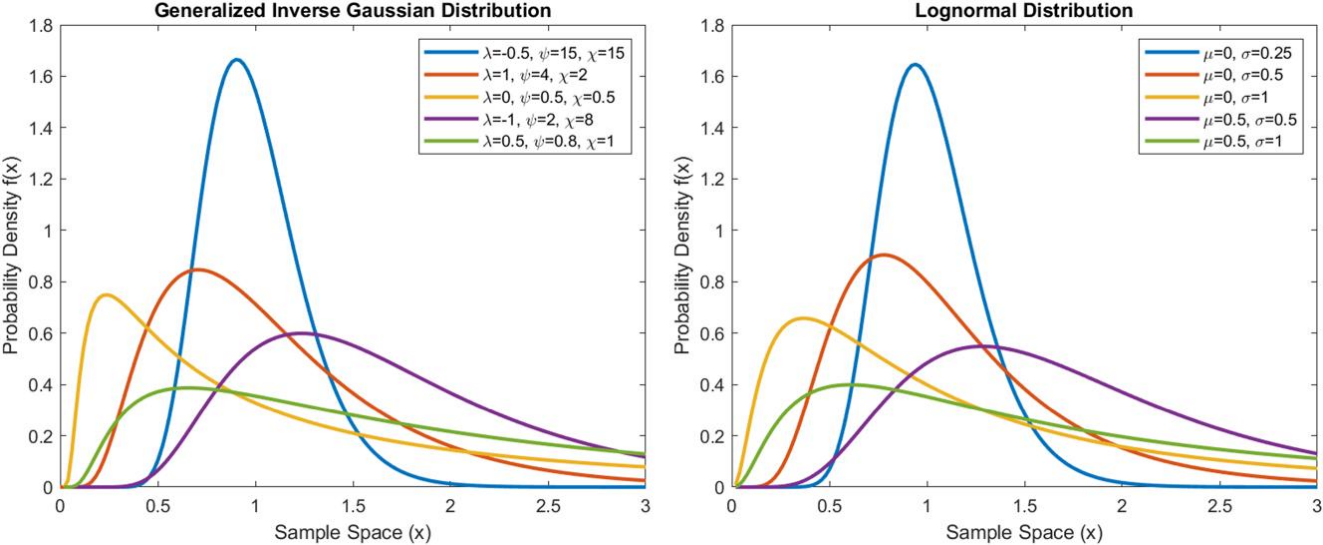
Examples of the generalized inverse Gaussian (GIG) and lognormal models with different parameter values. The GIG model and lognormal model can have similar shapes with certain parameter values.

The GIG model is defined as the following:


(1) $$f(x|\psi ,\;\chi ,\;\lambda )=\frac{{\left(\frac{\psi }{\chi }\right)}^{\lambda /2}}{2K_{\lambda }\left({\left(\frac{\psi }{\chi }\right)}^{1/2}\right)}x^{\lambda -1}\exp\;\left(-\frac{1}{2}\left(\psi x+\frac{\chi }{x}\right)\right),$$

where *−∞ < λ < ∞*, *ψ ≥* 0, *χ ≥* 0, and $K_{\lambda }$ is the modified Bessel function of the third kind with index *λ* [26]. *λ* determines the shape or skewness of the function, which can be categorized into 2 types. When λ is less than or equal to 0, the formula gives diffusion models, including the inverse Gaussian (IG) model (when $\lambda \;\;=\;\;-\frac{1}{2}$). When λ is greater than 0, the formula gives nondiffusion models, including the gamma model when *χ* → 0. The exponential model is a special case of the gamma model with *λ* = 1. *ψ* and *χ* together determine the concentration and scale of the function. $\sqrt{\psi \chi }$ is the concentration parameter that decides how concentrated the distribution is around its mean, and $\sqrt{\chi /\psi }$ is the scale parameter that decides the dispersion of the distribution [26]. Example plots of the GIG models are shown in Fig. 1.

The lognormal model is defined as


(2) $$f(x|\mu ,\;\sigma )=\frac{1}{x\sigma \sqrt{2\pi }}\exp\;\left(-\frac{{\left(\ln\;x-\mu \right)}^{2}}{2\sigma^{2}}\right),$$

where *−∞ < µ < ∞* and *σ* are greater than 0 [26]. Example lognormal plots are shown in Fig. 1.

### State-Space Model of Leptin Secretion

**Figure 2.**
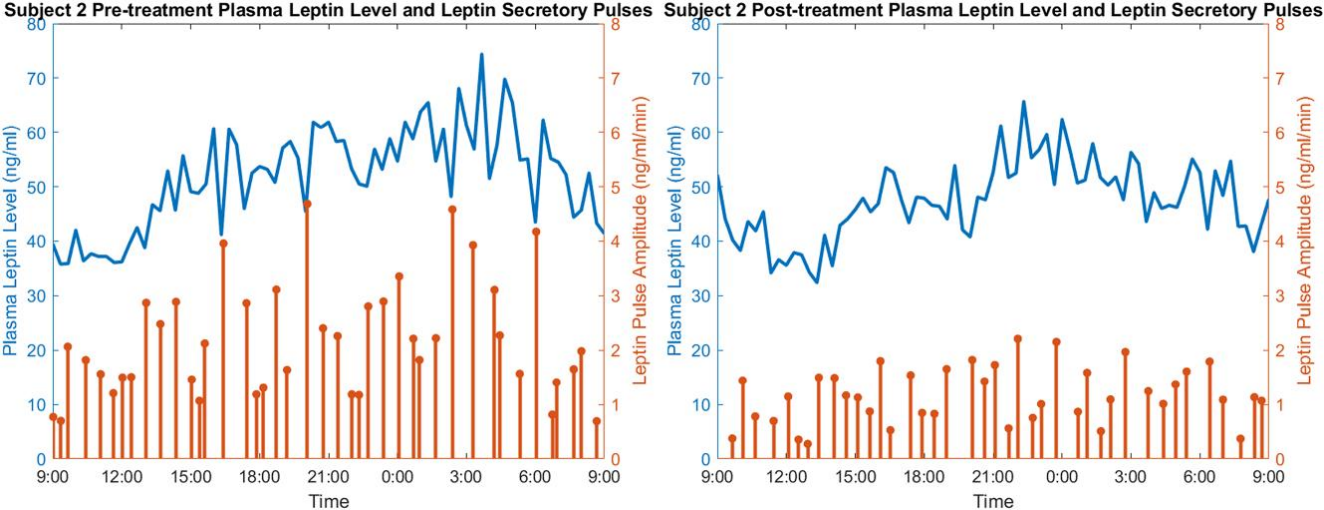
Example plasma leptin concentration level (line plot) and the extracted pulses (stem plot). An increase in plasma leptin level can be observed in both figures immediately following each pulse, especially ones with large amplitudes.

In their previous study, Reddy et al [17] extracted the leptin secretory pulses by deconvolving the plasma leptin level over time. The result shows the rate of leptin released into the blood at each time step. They assumed that the plasma leptin level can be modeled by a state-space model with two first-order differential equations representing the concentration of leptin in adipose and blood, respectively. The overall model is given by the following:


(3) $$\frac{dx_{1}(t)}{dt}=-\theta_{1}x_{1}(t)+u(t)\;\;\;\;(\mathrm{Adipose}\;\mathrm{tissue})\;$$

(4) $$\frac{dx_{2}(t)}{dt}=\theta_{1}x_{1}(t)-\theta_{2}x_{2}(t)\;(\mathrm{Plasma})\;$$

(5) $$y(t_{i})=x_{2}(t_{i})+\nu (t_{i}),\;\;\;\;\;i=1,\;\ldots ,\;N\;$$

where *x*_1_ and *x*_2_ are respectively the leptin levels in adipose and plasma at a given time, *N* is the number of measurements, and *y*(*t_i_*) is the measurement of plasma leptin level at time *t_i_*. Note that *u*(*t*) is a sequence of leptin secretory pulses produced in adipose tissue at time *t*, and *ν*(*t_i_*) models observation noise. The concentration of leptin in the adipose tissue at any time instant is the amount of leptin produced minus the amount of leptin diffused into the blood, and the concentration of leptin in the plasma is the infused leptin from adipose tissue minus the amount of leptin cleared. Through an optimization process, they determined both the infusion rate constant *θ*_1_ and the clearance rate constant *θ*_2_, as well as the secretory pulse process *u* that shows both the amplitude and the timing of each pulse. Since there are 18 participants each with pretreatment and posttreatment measurements, a total of 36 leptin secretory pulse event processes were extracted after deconvolution. Fig. 2 illustrates 2 examples of the extracted pulses with the original plasma leptin levels.

### Distribution Model Fitting

In this study, we further processed the data: For each extracted process, we created 2 vectors. The first vector represents the time intervals between successive pulse occurrences, or the interpulse intervals, ignoring the amplitudes. The second vector contains the amplitudes of the pulses, ignoring the timings. In our data set, the number of pulses per process ranges from 24 to 45, which is significantly fewer than the 97 to 348 pulses found in the previously mentioned EDA study [18].

After obtaining the interpulse intervals for each process, we fit each of the mentioned models to them. Specifically, we initialized the parameters and their upper and lower limits and then maximized the log-likelihood for each model. We set boundaries for the GIG model to distinguish between diffusion, nondiffusion, and gamma models. The parameter boundaries were set as described previously in this paper: The lower bounds for *ψ* and *χ* are 0 and their upper bounds are infinity. *λ* is from negative infinity to 0 for the diffusion model, and 0 to infinity for the nondiffusion model. It is worth noting that when *λ* = 0, it is considered a diffusion model, so we made sure the estimated nondiffusion model has strictly positive *λ*. Based on the boundaries and the meanings of the parameters, we finally decided on a set of initial parameters. For the diffusion and nondiffusion models, *ψ* and *χ* should be positive, and we chose to initialize both at 1. The sign of *λ* differentiates between diffusion and nondiffusion models, and we set *λ* to −1 and 1, respectively, for these models. As a special case, the gamma model has a simpler form:


(6) $$f(x|\gamma ,\;\beta )=\frac{1}{\beta^{\gamma }\Gamma (\gamma )}x^{\gamma -1}e^{-x/\beta },$$

where *γ* is greater than 0, β is greater than 0, and Γ is the gamma function. *γ* is the shape parameter, and β is the scale parameter [29]. Note that when *χ* → 0, equation (1) becomes equation (6) with *λ* set to *γ* and *ψ* set to $\frac{2}{\beta }$. With this widely used form of gamma distribution with 2 parameters, the optimization process becomes easier. The lognormal distribution has 2 parameters, *µ* and *σ*, which represent the mean and SD of the natural logarithm of the random variable, respectively [30]. Thus, we set *µ* and *σ* to their ideal values: the mean and SD of the natural logarithm of the data, respectively. These initial parameter settings were aimed at expediting the optimization process, but after experimenting with different initial guesses, we found them not to be particularly important in our fitting process. However, these considerations could be crucial for other model-fitting problems involving larger data sets or more sophisticated algorithms.

As used in the EDA study [18], we fit the models by maximizing the log-likelihood, which is the sum of the natural logarithm of the probability density of each interpulse interval in a process. To find the parameters that maximize the log-likelihood, we used MATLAB's *fmincon* function to minimize the negative log-likelihood. It returns a set of variables that minimizes the value of a nonlinear multivariable function within prescribed lower and upper bounds for the variables. In our case, the input function is the negative log-likelihood, and the variables are the parameters whose initialization and boundaries are discussed previously. We used the interior-point method for optimization [31].

We applied a similar method for fitting the amplitudes of the extracted pulses but with different models. We considered the Gaussian, lognormal, and gamma distribution models to be the candidates for the best fit of the amplitudes. Compared to the models used for the time intervals, we retained only the lognormal and gamma models and added the commonly used Gaussian model. We did not use the diffusion and nondiffusion GIG models because their complex nature lacked justification in this context. It is worth noting that the gamma model is part of the GIG family but with fewer parameters. Moreover, the lognormal model is a suitable choice for strictly positive data, which holds for the amplitudes [32].

### Model Evaluation and Comparison

After fitting the models and recording the estimated parameters, we evaluated and compared the performances of all models for each extracted process. For each model fit to each process, we computed the AIC defined as


(7) $$AIC=-2\log\;f({\hat{\theta }}_{ML})+2p,$$

where ${\hat{\theta }}_{ML}$ is the estimated set of parameters that maximize the likelihood, log *f* (${\hat{\theta }}_{ML}$) is the log-likelihood evaluated at ${\hat{\theta }}_{ML}$, and *p* is the number of parameters. A higher maximum log-likelihood and a smaller number of parameters mean better performance, so a lower AIC indicates a better fit [18].

**Figure 3.**
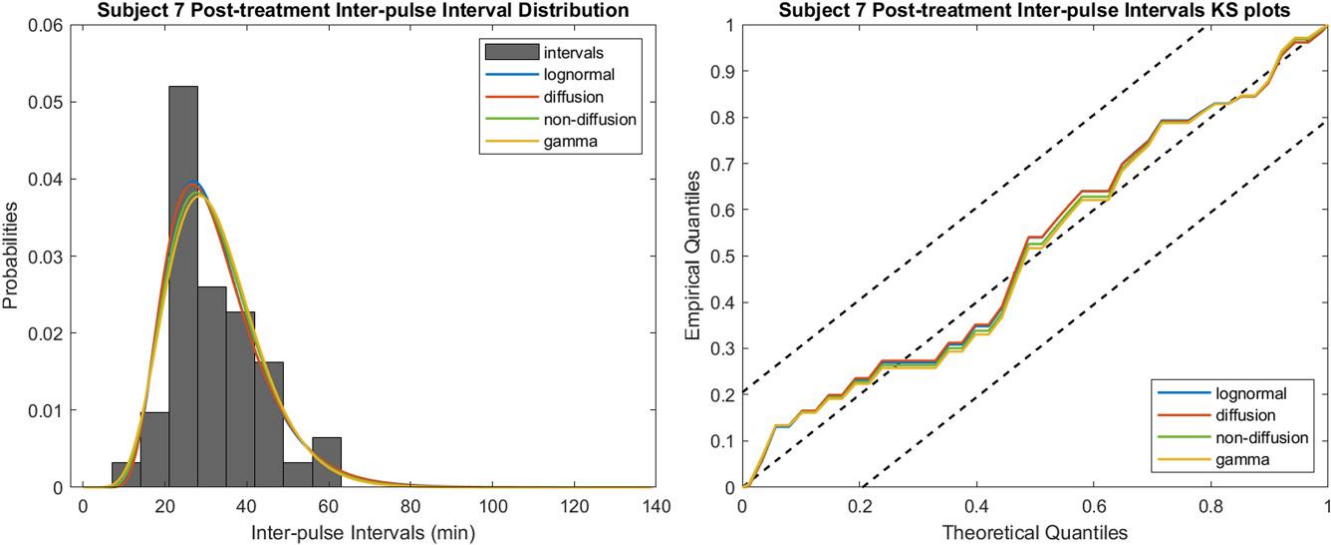
Example distribution of interpulse intervals with the estimated models and the corresponding Kolmogorov-Smirnov (KS) plots. The closeness between the estimated distribution models and the empirical histogram can be observed directly and is also indicated by the closeness between the KS plots and the line from (0, 0) to (1, 1). The upper and lower dashed lines are the boundaries of the 95% CI.

We also created KS plots as employed in the EDA study [18]. Making the KS plots involves applying the time-rescaling theorem [33]. This theorem states that any point process can be transformed into an exponential distribution with a rate of 1, using its conditional intensity function. If the rescaled times adhere to such an exponential distribution, they can be further transformed into uniform random variables. In a KS plot, these uniform random variables are sorted and plotted against the cumulative density function of a uniform distribution. Ideally, if the model is perfect, the plot should be a straight line from (0, 0) to (1, 1). We also incorporated a 95% CI, where exceeding this interval indicates a relatively poor fit (there is a 5% chance that the empirical data exceed this interval if they are from the same distribution of the theoretical data). We applied the method from [34] to find the critical value of the KS test *d_α_* that is needed to construct the CI. In addition, we computed the KS distance defined as the maximum distance between the empirical plot and the ideal line. Thus, a smaller KS distance signifies a better fit [33]. The steps to perform time-rescaling and create a KS plot and its CI for an estimated model can be found in the supplementary materials [35]. The estimated models for one sample and the corresponding KS plots are illustrated in Fig. 3.

For amplitudes, we also computed the AIC to evaluate the goodness of fit and used Q-Q plots with a 95% CI. We plotted the quantiles of the estimated models against the quantiles of the empirical data. Specifically, we calculated the inverse of the cumulative density function of the estimated models for each percentage to obtain the theoretical quantiles. We then computed the empirical quantiles at the same percentages. If the estimated and empirical data are from the same distribution, the resulting plot will be linear with a slope equal to 1. The results of the distribution fitting of amplitudes and the Q-Q plots are included in the supplementary materials [35].

## Results

The computed AIC scores for each model across all participants are shown in Table 1. The scores are very similar, as indicated in the graph. This similarity is also true for the rest of the data set and is reflected by the model plots as well. This could be a result of using the same cost function and optimization method in our fitting process. The final costs of the models included in the AIC calculation could be close due to similar stopping criteria for the algorithm. Thus, the number of parameters plays an important role in the evaluation. With one fewer parameter, the best model for each process is typically either the lognormal or the gamma model. However, it is noteworthy that the diffusion model occasionally outperforms the others despite having more parameters than the lognormal and gamma models. Although the AIC scores are smaller than those in the EDA study in general, it does not suggest that our models have better fitting performances because AIC scores do not have any intrinsic value [36]. It is used to compare models that are fit to the same sample.

**Table 1.** Akaike's information criterion of all interpulse interval distribution models for each sample

Sample	Mean interval, min	Lognormal	Diffusion	Nondiffusion	Gamma
Subject 1-pre	37.05	285	**285**	287	288
Subject 1-post	37.11	307	**307**	309	311
Subject 2-pre	34.63	336	337	334	**332**
Subject 2-post	35.46	**293**	293	295	295
Subject 3-pre	34.07	**319**	320	321	322
Subject 3-post	34.05	303	305	304	**302**
Subject 4-pre	36.33	**320**	323	323	321
Subject 4-post	34.10	**317**	319	319	317
Subject 5-pre	36.33	**295**	295	297	297
Subject 5-post	39.51	285	**284**	288	291
Subject 6-pre	38.78	298	**294**	301	307
Subject 6-post	32.32	340	342	339	**337**
Subject 7-pre	45.10	**267**	268	268	267
Subject 7-post	32.30	337	339	339	**337**
Subject 8-pre	34.52	**322**	323	323	323
Subject 8-post	37.84	294	296	296	**294**
Subject 9-pre	31.79	344	348	342	**340**
Subject 9-post	35.79	**310**	312	312	310
Subject 10-pre	42.50	**270**	271	272	272
Subject 10-post	39.97	290	291	291	**289**
Subject 11-pre	37.78	**293**	293	295	295
Subject 11-post	40.76	281	284	282	**280**
Subject 12-pre	34.56	**323**	324	325	325
Subject 12-post	34.55	**317**	319	319	317
Subject 13-pre	35.79	**304**	305	305	304
Subject 13-post	33.66	330	331	331	**329**
Subject 14-pre	35.76	**292**	293	294	295
Subject 14-post	54.36	221	224	222	**220**
Subject 15-pre	31.86	348	351	344	**342**
Subject 15-post	35.12	**331**	333	333	332
Subject 16-pre	36.18	310	313	311	**309**
Subject 16-post	35.74	**320**	322	322	320
Subject 17-pre	50.00	**247**	248	249	249
Subject 17-post	57.39	215	217	215	**213**
Subject 18-pre	35.46	**313**	316	316	315
Subject 18-post	35.92	**301**	303	303	302

A lower AIC suggests a better fit. The best model for each process is in bold.

The KS distances for these 4 models are also quite similar, with most falling within the 95% CIs. The nondiffusion model emerges as the best model in only 1 case, while the gamma model, a special case of the nondiffusion model, is the best in 16 instances. The diffusion and lognormal models rank as the best in other cases. The computed KS distances for each model are presented in Table 2. Given the limited size of our data set, it is challenging to definitively determine which model is the best overall.

**Table 2.** Kolmogorov-Smirnov distances of all interpulse interval distribution models for each sample with a 95% significance cutoff

Sample	Mean interval, min	Lognormal	Diffusion	Nondiffusion	Gamma
Subject 1-pre	37.05	0.127*^a^*	**0.118** *^a^*	0.129*^a^*	0.135*^a^*
Subject 1-post	37.11	0.173*^a^*	**0**.**163***^a^*	0.177*^a^*	0.183*^a^*
Subject 2-pre	34.63	0.220	0.226	0.204*^a^*	**0**.**204***^a^*
Subject 2-post	35.46	0.085*^a^*	**0**.**068***^a^*	0.087*^a^*	0.102*^a^*
Subject 3-pre	34.07	**0**.**102***^a^*	0.109*^a^*	0.103*^a^*	0.112*^a^*
Subject 3-post	34.05	0.127*^a^*	0.131*^a^*	0.123*^a^*	**0**.**123***^a^*
Subject 4-pre	36.33	0.088*^a^*	0.095*^a^*	0.091*^a^*	**0**.**087***^a^*
Subject 4-post	34.10	**0**.**085***^a^*	0.088*^a^*	0.088*^a^*	0.087*^a^*
Subject 5-pre	36.33	0.107*^a^*	**0**.**091***^a^*	0.111*^a^*	0.126*^a^*
Subject 5-post	39.51	0.079*^a^*	**0**.**061***^a^*	0.088*^a^*	0.109*^a^*
Subject 6-pre	38.78	0.179*^a^*	**0**.**148***^a^*	0.199*^a^*	0.222*^a^*
Subject 6-post	32.32	0.097*^a^*	0.104*^a^*	0.077*^a^*	**0**.**077***^a^*
Subject 7-pre	45.10	0.127*^a^*	0.125*^a^*	0.124*^a^*	**0**.**119***^a^*
Subject 7-post	32.30	**0**.**077***^a^*	0.077*^a^*	0.082*^a^*	0.090*^a^*
Subject 8-pre	34.52	0.143*^a^*	0.146*^a^*	0.141*^a^*	**0**.**122***^a^*
Subject 8-post	37.84	0.106*^a^*	0.106*^a^*	0.098*^a^*	**0**.**094***^a^*
Subject 9-pre	31.79	0.133*^a^*	0.150*^a^*	0.117*^a^*	**0**.**117***^a^*
Subject 9-post	35.79	0.121*^a^*	0.121*^a^*	0.120*^a^*	**0**.**116***^a^*
Subject 10-pre	42.50	**0**.**142***^a^*	0.144*^a^*	0.144*^a^*	0.168*^a^*
Subject 10-post	39.97	0.175*^a^*	0.178*^a^*	0.160*^a^*	**0**.**160***^a^*
Subject 11-pre	37.78	0.116*^a^*	**0**.**114***^a^*	0.117*^a^*	0.116*^a^*
Subject 11-post	40.76	0.139*^a^*	**0**.**138***^a^*	0.145*^a^*	0.145*^a^*
Subject 12-pre	34.56	0.117*^a^*	**0**.**108***^a^*	0.117*^a^*	0.123*^a^*
Subject 12-post	34.55	0.093*^a^*	0.097*^a^*	**0**.**093***^a^*	0.104*^a^*
Subject 13-pre	35.79	0.097*^a^*	**0**.**095***^a^*	0.096*^a^*	0.100*^a^*
Subject 13-post	33.66	0.200*^a^*	0.201*^a^*	0.189*^a^*	**0**.**183***^a^*
Subject 14-pre	35.76	**0**.**064***^a^*	0.069*^a^*	0.065*^a^*	0.077*^a^*
Subject 14-post	54.36	0.122*^a^*	0.124*^a^*	0.107*^a^*	**0**.**107***^a^*
Subject 15-pre	31.86	0.144*^a^*	0.155*^a^*	0.127*^a^*	**0**.**127***^a^*
Subject 15-post	35.12	0.141*^a^*	0.139*^a^*	0.134*^a^*	**0**.**120***^a^*
Subject 16-pre	36.18	**0**.**082***^a^*	0.083*^a^*	0.092*^a^*	0.092*^a^*
Subject 16-post	35.74	0.135*^a^*	0.133*^a^*	0.127*^a^*	**0**.**117***^a^*
Subject 17-pre	50.00	0.155*^a^*	**0**.**132***^a^*	0.159*^a^*	0.181*^a^*
Subject 17-post	57.39	0.198*^a^*	0.204*^a^*	0.177*^a^*	**0**.**177***^a^*
Subject 18-pre	35.46	**0**.**129***^a^*	0.135*^a^*	0.135*^a^*	0.130*^a^*
Subject 18-post	35.92	0.105*^a^*	**0**.**102***^a^*	0.105*^a^*	0.108*^a^*

A smaller KS distance suggests a better fit. *^a^* The best model for each sample is in bold, models within the significance cutoff are marked with.

**Figure 4.**
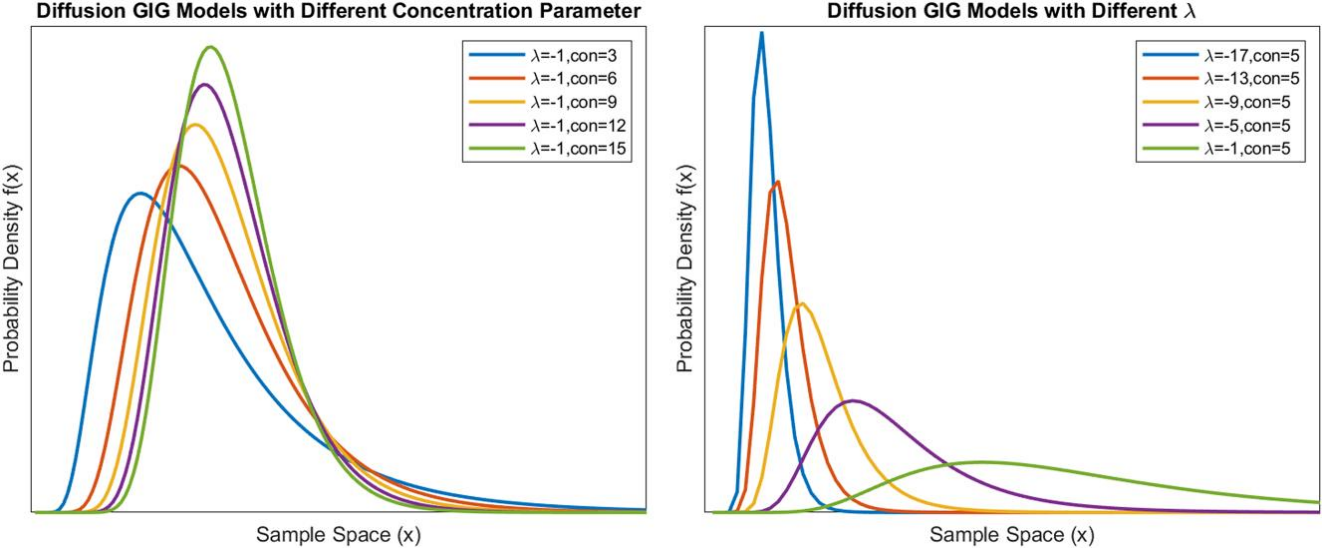
Demonstration of effects of parameters in the diffusion generalized inverse Gaussian (GIG) model. The graphs show the effects of a changing *λ* or a changing concentration parameter. In both graphs, the scale parameter $\sqrt{\chi /\psi }=1$, and a concentration shift to the right is present. “Con” stands for the concentration parameter $\sqrt{\psi \chi }$.

There are instances, such as the KS distances for subject 2, where the posttreatment data scores are lower, indicating a better fit. However, the scores in these cases generally vary from one another. We also calculated the parameter change in all the models for both interpulse intervals and amplitude distributions. We applied the Wilcoxon signed rank test [37] to test the null hypothesis that the change amounts for a parameter across the participants come from a distribution with a mean equal to zero. With a 5% significance level, we failed to reject the null hypothesis for almost all parameters of all models. The only 2 exceptions were with *λ* and the concentration parameter $\sqrt{\psi \chi }$ [26, 38, 39] of the diffusion model for fitting interpulse intervals. In both cases, the differences in parameters were mostly positive, indicating an increase after bromocriptine treatment. Fig. 4 demonstrates the effects of increasing *λ* and the concentration parameter in a diffusion model. This might suggest that the posttreatment interpulse intervals distribution is concentrated further from 0 than the pretreatment distribution.

**Figure 5.**
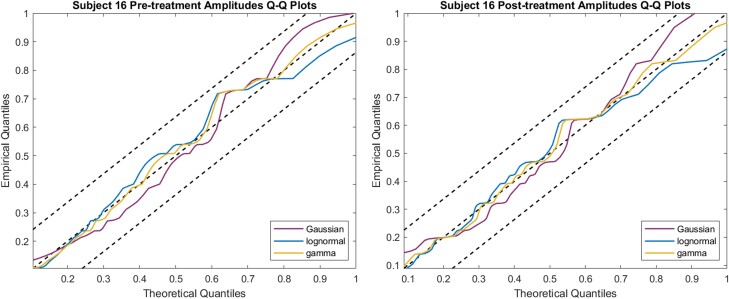
Example Q-Q plot of pulse amplitudes vs models. The models are Gaussian, lognormal, and gamma distributions. The dashed line in the middle is the reference line indicating the ideal case in which the two distributions are the same. The upper and lower dashed lines are the boundaries of the 95% CI.

For the fitted models of pulse amplitudes, we computed their AIC and corresponding Q-Q plots, and they showed little difference between them. In the case of subject 16, as shown in Fig. 5, the gamma model captures the distribution slightly better with both pretreatment and posttreatment data than the other 2 models as its Q-Q plot is the closest to the reference line in both, but that is not always the case for all participants. In all 36 cases, the correlation coefficients between theoretical and empirical quantiles are above 0.9, indicating a strong similarity between the two and thus suggesting a dominant general pattern that can describe the behavior of leptin secretion in terms of pulse amplitudes. Tables of the AIC and the correlation coefficients for the pulse amplitudes are included in the supplementary materials [35].

## Discussion

Multiple factors contribute to leptin synthesis, such as body fat, food intake, and insulin [19, 40]. These factors are not discernible from the extracted pulses, and the deconvolution process used for pulse sequence reconstruction might have caused information loss. Therefore, we chose the 4 more complex models mentioned previously and did not consider a one-parameter exponential model. While model complexity is necessary to reflect the underlying leptin synthesis, both the gamma and lognormal models, each with 2 parameters, yielded AIC and KS distance scores comparable to models with 3 parameters. Additionally, leptin synthesis occurs at multiple sites, and our data were derived from plasma leptin levels that were likely an aggregation of leptin secretion from countless such sites [19].

This implies a need for a mixture of models in depicting leptin secretion for the distribution of the interpulse intervals. Nonetheless, our results show that each of our models alone could capture the behavior of secretion timings, and no model performed better than others consistently.

In addition to the interpulse intervals, the distributions of the amplitudes also reflected potential features of the physiology of leptin. First, the distribution is bell shaped, indicating that extremely low or high amplitude pulses are unlikely, a pattern expected in healthy individuals. Second, the distribution is nearly symmetric, as evidenced by its representation through a Gaussian distribution. However, the skew to the right in both the lognormal and gamma models, along with their comparable fit in terms of the AIC and quantile analysis, suggests that smaller pulses are more common, while larger pulses are rarer and more extreme. This pattern might be a result of leptin being secreted into the blood from various fat cells and not being perfectly synchronized. Additionally, when secretory events occur closely in time, they might have been recorded as a large increase in plasma leptin level at one measurement. The deconvolved pulse sequence might have treated these real secretory pulses as one large pulse.

In both the interpulse intervals and pulse amplitude analyses, there was no apparent pattern of change due to bromocriptine intake. By using the Wilcoxon signed rank test on the change of parameters, we failed to reject the null hypothesis that the differences in all the amplitude models and most of the interpulse intervals models come from a zero median distribution. However, we found an increase in *λ* (*P* = .039) and the concentration parameter (*P* = .028) in the diffusion model. The difficulty in finding an apparent effect could be caused by leptin's dependencies on various factors in the human body, as mentioned earlier. Studies have also found that leptin levels in plasma can be influenced by external factors such as exposure to cold and stress [41, 42], which could have affected the measurements. Nonetheless, the concentration of the diffusion model shifting to the right might suggest a decrease in the number of extremely short intervals after bromocriptine treatment. In addition, as demonstrated in Fig. 4 the increase of the concentration parameter generally means a more concentrated distribution around the mean and lighter tails on both ends of the curve, suggesting a more regulated leptin secretion pattern with a smaller variation in the lengths of the interpulse intervals. However, this possible implication based on the increase of the concentration parameter is undermined by the increase of *λ*, which causes the estimated distribution to be more dispersed, as shown in Fig. 4. In addition, the fact that only the diffusion model for interpulse intervals reflected a pattern of change according to the statistical test might indicate that it is the best model among all the ones used in this study, which conforms with the assumption that the leptin secretion process involves an integrate-and-fire mechanism.

To conclude, the results of this study show the effectiveness of modeling pulsatile hormone secretion using the mentioned probability models, which could be used to characterize other similar endocrine processes. Although we have not found any statistically significant effect of bromocriptine by observing the model parameters before and after treatment, we have presented a new method of testing the influence of a treatment on a secretion process. The estimated distribution models had similar performances according to the implemented metrics. For modeling the interpulse intervals, the diffusion model's usefulness is backed by its connection with the integrate-and-fire mechanism, whereas the gamma model had the best scores in most cases. The nondiffusion model performed slightly worse in most cases but could still be a plausible choice in similar future studies. For modeling the pulse amplitudes, all tested distribution models had comparable performances with gamma being the best model in most cases. Further investigation may include tail analysis to compare these distribution models. In addition, this study investigated only 18 women, whereas a larger group of individuals and a longer observation time might reveal more information about the underlying mechanism of leptin secretion. To continue the research of leptin's physiology as an effort to develop better treatment for obesity, the next step could be a comparison between the data and results analyzed in this study and those of individuals without obesity. This may contribute to a better characterization of the influence of obesity on leptin secretion. Researchers may also employ the methods in this study to examine other hormone secretion dynamics, explore the effects of external factors such as different medicines and illnesses, and design regulation protocols based on the results.

## Data Availability

Data analyzed during this study are included as figures in this article and its supplementary information as provided in a data repository, including plasma leptin measurements and secretory pulse information.

## Abbreviations

AIC: Akaike’s information criterion
EDA: electrodermal activity
GIG: generalized inverse Gaussian
IG: inverse Gaussian
KS: Kolmogorov-Smirnov
